# Prophylactic and Therapeutic Efficacy of Prebiotic Supplementation against Intestinal Coccidiosis in Rabbits

**DOI:** 10.3390/ani9110965

**Published:** 2019-11-13

**Authors:** Saeed A. El-Ashram, Shawky M. Aboelhadid, El-Sayed M. Abdel-Kafy, Shymaa A. Hashem, Lilian N. Mahrous, Eman M. Farghly, Usama K. Moawad, Asmaa A. Kamel

**Affiliations:** 1 College of Life Science and Engineering, Foshan University, Foshan 528231, Guangdong, China; 2 Faculty of Science, Kafrelsheikh University, Kafr El-Sheikh 33516, Egypt; 3 Parasitology Department, Faculty of Veterinary Medicine, Beni Suef University, Beni-Suef 62511, Egypt; dr.lilian_nagy@yahoo.com (L.N.M.); drasmaaalaa@yahoo.com (A.A.K.); 4 Animal Production Research Institute, Agricultural Research Center, Dokki, Giza 12651, Egypt; sayedabdkaffy@yahoo.com (E.-S.M.A.-K.); shaimaaahmedparavet@yahoo.com (S.A.H.); hawk18922@gmail.com (E.M.F.); 5 Department of Histology and Cytology, Faculty of Veterinary Medicine, Beni-Suef University, Beni-Suef 62511, Egypt; dr.usamakm2013@yahoo.com

**Keywords:** rabbits, intestinal *Eimeria* species, prebiotic, histopathological findings, biochemical parameters

## Abstract

**Simple Summary:**

There are limitations for using chemical products in meat production. The use of prebiotics to control *Eimeria* infections in rabbits may be of value. Prebiotics as a prophylaxis resulted in diminishing adverse effects caused by *Eimeria* spp. through decreasing fecal oocyst counts, retaining body weight, and reducing the number of parasitic stages in the intestinal tissues when compared with the non-supplemented controls.

**Abstract:**

This study was conducted to investigate the effect of prebiotic supplementation against intestinal coccidiosis in rabbits. Fifty male rabbits aged 35–60 days (1–1.5 kg) were divided into prophylactic and therapeutic experiments (five groups, 10 rabbits per group). Prophylactic experiment had prebiotic supplemented (PS-P), non-supplemented infected control (NI-P), and non-supplemented non-infected control (NN-P) groups. Ten days post-prebiotic supplementation (PPS), rabbits in groups PS-P and NI-P were infected orally with 5.0 × 10^4^ sporulated oocysts of mixed *Eimeria* species. However, therapeutic experiment had prebiotic supplemented (PS-T) and untreated infected (UI-T) groups of naturally infected rabbits with *Eimeria* species. A significant reduction in oocyst count per gram feces (OPG) (*p* ≤ 0.05) was reported in the PS-P (57.33 × 10^3^ ± 2.84) and NI-P (130.83 × 10^3^ ± 43.38) groups during the experiment. Additionally, rabbits in groups (PS-P, 970.33 ± 31.79 g and NI-P, 870.66 ± 6.66 g) showed weight loss after infection. However, a significant (*p* ≤ 0.05) decrease in OPG was observed at day seven PPS in the PS-T group (4 × 10^3^ ± 0.00) when compared with the UI-T group (32 × 10^3^ ± 7.54). Furthermore, the PS-T group had a higher body weight than rabbits in the UI-T group. Histopathological findings of the intestinal tissues (duodenum, jejunum, and ileum) showed that the counts of the endogenous stages were significantly higher in the NI-P and UI-T groups than in the prebiotic-supplemented groups (PS-P and PS-T). Supplementation of the prebiotic did not have any adverse effects on biochemical parameters, such as AST, ALT, creatinine, total protein, and total cholesterol. In conclusion, prebiotic supplementation can be used to minimize the adverse effects of intestinal coccidiosis in rabbits, which in turn limits body weight loss, especially for the prophylaxis of coccidial infection.

## 1. Introduction

Coccidiosis is a parasitic disease that causes severe economic losses in rabbit production [1,2]. Rabbit coccidiosis is caused by thirteen species of the genus *Eimeria* [3]. There are two types of rabbit coccidiosis intestinal (*Eimeria perforans*, *E. magna*, *E. media*, and *E. irresidua*) and hepatic (*E. steidae*) [4,5,6,7]. Intestinal coccidiosis in rabbits is associated with diarrhea, dehydration, inappetence, and weight loss [3,8]. Rabbits between the ages of one and three months are most susceptible to coccidiosis, especially after weaning [9,10]. Mother rabbits are generally more susceptible to infection during the perinatal period and before weaning [10,11]. Several control strategies have been applied to treat and prevent coccidiosis. However, anticoccidial drugs remain the most common agents that have been utilized in the control of rabbit coccidiosis. Anticoccidial drugs have been used as feed or water supplements to control coccidiosis; however, the increase in resistance to many of these products and chemical residues in meat products has raised concerns about the need for new alternatives for the control of coccidial infections [3]. New alternatives are emerging, including anticoccidials obtained from plants, fungi, or microorganisms. Alternative anticoccidials have the potential to inhibit the growth of pathogenic micro-organisms, improve the immune system, and increase the animal productivity [3,8]. Prebiotics are nondigestible food components that promote the growth of beneficial bacteria in the digestive system and host defense against infections [12,13,14,15]. Bio-Mos^®^, which has been used in the animal husbandry industry, shows suppressing effect on enteric pathogens. Bio-Mos^®^ plays an important role in modulating the immune response of chickens and turkeys [16,17], and enhances the growth and productivity of pigs [18]. Moreover, dietary supplementation of prebiotic and probiotic decreased the mortality and improved the adverse clinical signs in rabbits experimentally infected with *Pasterella multocida* [19]. Therefore, this study aimed to evaluate the prophylactic and therapeutic use of prebiotics against intestinal coccidiosis in rabbits.

## 2. Materials and Methods

This work was conducted according to the ethical standards of Faculty of Veterinary Medicine, Beni-Suef University, Egypt and approved by the Institutional Animal care and Use Committee of Beni-Suef University (2019-BSUV-39).

### 2.1. Experimental Rabbits 

Fifty weaned male rabbits (V-Line breed) aged 35–60 days and weighed 1–1.5 kg, were used in this study. Rabbits were separated from their mothers, and each rabbit was placed in a separate wire mesh cage. Rabbits were weaned in a separate cage away from mothers in a pen. The pen was pyramidal in shape and double sided, with two levels. The lower one was one m above the pen floor. Each level had 10 wire cages, each with dimensions of 50 cm × 50 cm × 30 cm. Each cage contained a foot bad to protect their feet. Rabbits fed on commercial rabbit pelleted diet (free from anticoccidial drugs) via feeding hoppers of galvanized steel. Water was provided by pottery drinkers. The feed and water were ad libitum. Rabbits were maintained at a constant 22 °C on a 12-h light-dark cycle in cages. Rabbits were individually housed in cages to collect feces. Each rabbit was numbered by ear and cage tags. All rabbits were weighed, and fecal samples were collected and examined by fecal floatation test [20,21,22] to confirm the absence of *Eimeria* infection upon arrival. 

### 2.2. Prebiotic Product 

Prebiotic product (Bio-Mos^®^), which was used in this study, was manufactured by ALLTECH, INC.CO., Nicholasville, KY, USA. Each 1 kg was composed of *Saccharomyces cerevisiae* cell wall (800 g), Mannan oligosaccharides (56 g), and dried *Saccharomyces cerevisiae* fermentation solubles (200 g).

### 2.3. Preparation of Eimeria Species Oocysts 

*Eimeria* spp. oocysts were obtained from the fecal samples of naturally infected rabbits. Samples were processed using a modified McMaster technique MAFF, 1986. Oocysts were transferred into 2.5% potassium dichromate solution at 27 °C with 60%–80% humidity for seven days [23,24,25]. Sporulated oocysts were centrifugally washed using distilled water and microscopically identified according to [3].

### 2.4. Experimental Design of Prebiotic Efficacy Against Coccidiosis

#### 2.4.1. Prophylactic Experiment 

A total of 30 rabbits were randomly allocated into three groups (10 rabbits each), including prebiotic supplemented (PS-P), non-supplemented infected control (NI-P), and non-supplemented non-infected control (NN-P) groups. Rabbits in the PS-P group were supplemented with prebiotic 2 g/L drinking water. NI-P and NN-P groups were served as positive and negative controls, respectively. Ten days post-prebiotic supplementation (PPS), rabbits in PS-P and NI -P groups were inoculated orally using a syringe with 5.0 × 10^4^ sporulated oocysts of mixed *Eimeria* species, including *E. media* (28%), *E. perforans* (17.14%), *E. intestinalis* (17.14%), *E. magna* (14.28%), *E. coecicola* (8.57%), *E. exigua* (7.14%), and *E. flavescens* (7.14%) for each rabbit. The prebiotic supplementation was continued until the end of the experiment. Fecal samples were examined daily until day 10 post-infection [2]. At day 10 post-infection, three rabbits from each group were slaughtered. Sera and intestinal tissue samples were collected for biochemical and histopathological analyses, respectively. 

#### 2.4.2. Therapeutic Trial 

Natural infection of intestinal coccidiosis in a rabbit farm was observed at day 48 of age. The clinical signs of coccidiosis were diarrhea, inappetence, bloating, and dehydration. The infection intensity was diagnosed using fecal oocyst counts. Rabbits with oocyst counts of ≤20,000 per gram feces had a confirmed rabbit coccidiosis [8]. Naturally infected rabbits (*n* = 20), which had nearly the same degree of infection depending on oocyst counts and body weight, were selected and divided into two groups of 10 rabbits each, including prebiotic supplemented (PS-T) and untreated infected (UI-T) groups. Rabbits in the PS-T group were treated by a prebiotic supplement at a dose of 2 g/L daily in drinking water for one week while the UI-T group did not receive any treatment. Fecal oocyst examination was assessed for up to one week PPS. Three representative rabbits from each group were slaughtered for histopathology examinations at day seven PPS. 

### 2.5. Evaluation Parameters in both Experiments

#### 2.5.1. Clinical Signs of Eimeria Infection in Rabbits

Clinical signs of rabbit coccidiosis were assessed according to a previously published method [3].

#### 2.5.2. Necropsy Examination

Three rabbits from each group were chosen randomly at the end of each experiment for macroscopic (gross) examination of the duodenum, jejunum, and ileum.

#### 2.5.3. Oocyst Counts Per Gram Feces (OPG)

Fecal samples were collected daily from each group, and OPG was assessed by McMaster technique. 

#### 2.5.4. Growth Rate

Body weight of rabbits was recorded at day zero and 10 post-infection. The body weight and weight gain in each group was determined by subtracting the body weight of the rabbits at the time of prebiotic supplementation or infection, from the body weight at the end of the experiment [26].

#### 2.5.5. Histopathological Examination

Specimens from different parts of duodenum, jejunum, ileum, and colon were fixed in 10% buffered formalin for histopathology. The fixed tissues were washed in running tap water over-night, dehydrated and infiltrated by paraffin wax. Serial paraffin sections (5 μm thickness) were obtained, and the sections were deparaffinized in three, consecutive washings in xylol for 5 min, and rehydrated with five, successive washings with alcohol in descending order of 100%, 95%, 80%, 70%, and 50% in deionized water. The histological sections were then subjected to conventional Hematoxylin and Eosin (H and E) staining procedure [27].

#### 2.5.6. Biochemical Parameters

Five mL of blood was collected in sterilized tubes during slaughtering of rabbits (prophylactic experiment) from the jugular vein of each rabbit. Tubes were centrifuged at 2500× *g* for 10 min, and sera were separated for biochemical analysis using an automatic clinical chemistry analyser [28]. Serum samples were analyzed for total proteins (TP), total cholesterol (TC), alanine amino transferase (ALT), alkaline phosphatase (ALP), aspartate amino transferase (AST), and creatinine.

### 2.6. Statistical Analysis 

Data were coded and entered using the statistical package for Social Sciences SPSS version 22. Data were analyzed using ANOVA tests and subsequent Duncan’s multiple range tests as well as the application of independent sample *t*-tests to determine the differences between means. Results were expressed as means ± SE. Probability values of less than 0.05 (*p* ≤ 0.05) were considered significant. 

## 3. Results

### 3.1. Prebiotic Efficacy as a Prophylaxis against Eimeria Species in Experimentally Infected Rabbits

#### 3.1.1. Clinical Signs of Coccidiosis in Rabbits

The clinical signs were less severe in the rabbits of the PS-P group than in the NI-P group. Rabbits in the PS-P group suffered from profound diarrhea (watery consistency of feces) with a decrease in feed intake, while rabbits in the NI-P group showed diarrhea with mucus, inappetence, bloating, rough hair, and dullness. The NN-P group had no clinical signs of disease.

#### 3.1.2. Post-Mortem Lesions

There were severe congestion, bloating, and mucoid contents, tinged with blood in different parts of intestinal tract in NI-P rabbits, while mild to moderate congestion with loose intestinal contents without bloating was marked in the prebiotic supplemented rabbits (PS-P). No signs of coccidial infection were recorded in NN-P rabbits.

#### 3.1.3. Oocysts Per Gram of Feces

**Figure 1:**
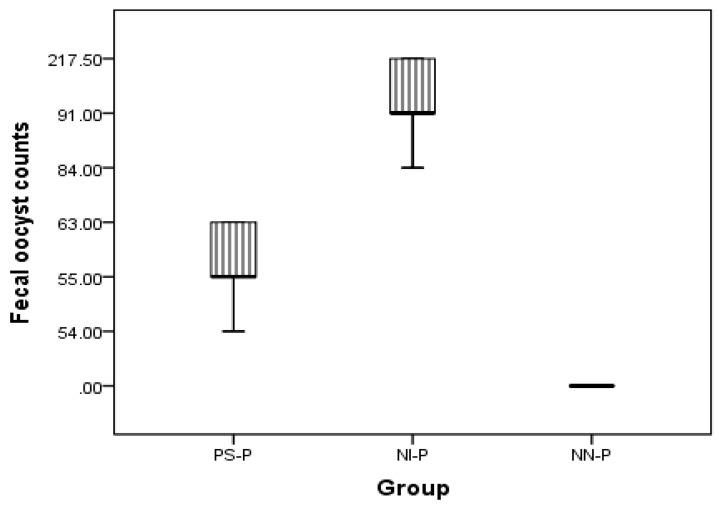
Oocyst counts at day 10 post-infection in the prophylactic experiment (PS-P, NI-P, and NN-P). PS-P = prebiotic supplemented, NI-P = non-supplemented infected control, and NN-P = non-supplemented non-infected control.

Oocyst excretion in the feces began at day five post-infection in both groups PS-P and NI-P. OPG was lower in the PS-P group (97.33 × 103 ± 19.63) than in the NI-P group (269 × 103 ± 50.78) (Table 1). This significant decrease in oocyst count in the PS-P group continued until end of the experiment (at day 10 post-infection) (Figure 1). 

#### 3.1.4. Body Weight of Rabbits

The PS-P group showed a significant (*p* ≤ 0.05) increase in the body weight (980.33 ± 2.88 g) as compared to the NI-P group (900.66 ± 4.66 g) before infection (Table 2). While in post-infection, both groups showed weight loss due to infection but the loss was somewhat limited in the PS-P group until day 10 of infection (Table 2). Similarly, the NN-P group had a higher body weight than rabbits in the PS-P and NI-P groups at day 10 post-infection.

### 3.2. Treatment of Coccidiosis in Naturally Infected Rabbits with Prebiotic Supplementation

#### 3.2.1. Oocyst Counts 

There was a significant (*p* ≤ 0.05) decrease in oocyst count (4 × 10^3^ ± 0.00) in rabbits supplemented with the prebiotic product (PS-T) at day seven PPS in comparison to that drenched water without the prebiotic product (UI-T) (32 × 10^3^ ± 7.54) (Table 3).

#### 3.2.2. Body Weight of Rabbits Supplemented with a Prebiotic Product 

There was no difference in body weight between the groups at the beginning of the treatments. However, the body weight loss became negligible in the PS-T group and continued in the UI-T group at day seven PPS (Table 4).

### 3.3. Histopathological Findings in Prophylactic and Therapeutic Experiments

**Figure 2:**
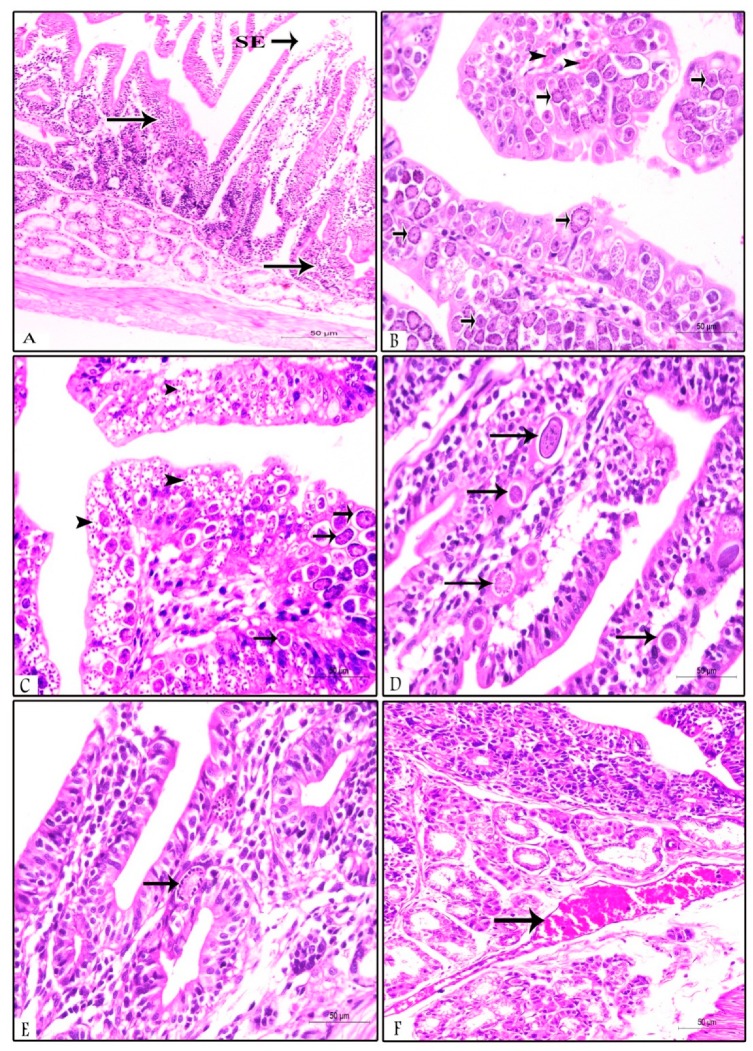
Duodenum in the NI-P group (non-supplemented infected control) showing: (**A**) Sloughing of the villous epithelium (SE) and massive mononuclear cell infiltration (arrows); (**B**) intestinal epithelium highly invaded by a huge number of different developmental stages of coccidial parasite (Note: Multifocal areas of discrete haemorrhages, arrow heads); (**C**) different developmental parasitic stages, including gametocytes and oocysts (arrows) in addition to multiple schizonts (arrow heads) occupying the sites of intestinal absorptive epithelium; (**D**) some coccidial stages in the lamina propria (arrows); (**E**) glandular epithelium contains parasitic stages (arrow); and (**F**) severe congestion of submucosal blood vessel (arrow). H&E stain, ×400.

**Figure 3:**
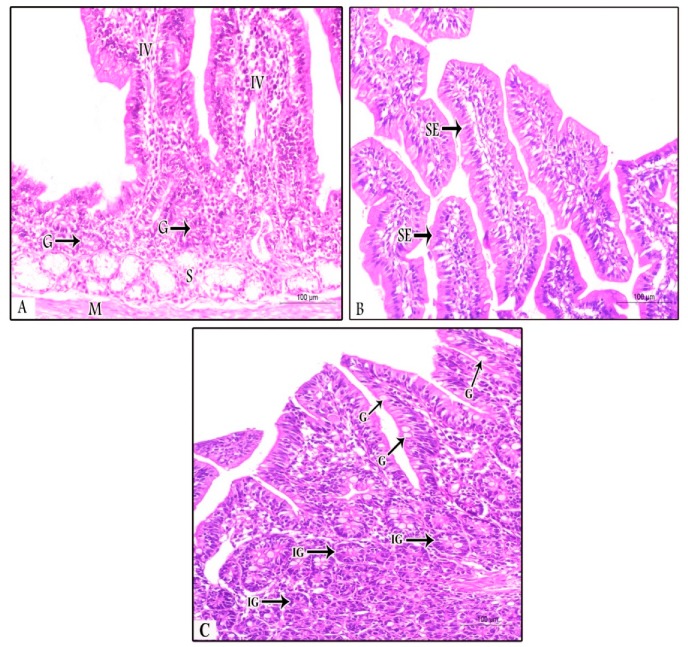
Dodenum in the NN-P group (non-supplemented non-infected control) showing: (**A**) Normal intestinal villi (IV) with their underlying lamina propria containing intestinal glands (G), submucosa (S), and muscularis mucosa (M) (H&E, ×200); (**B**) normal absorptive epithelium of simple columnar type (SE) lined the intestinal villi (H&E stain, ×200); and (**C**) intestinal villi lined with absorptive epithelium containing goblet cells (G) in between and normal intestinal glands (IG) in the lamina propria (H&E stain, ×200).

**Figure 4:**
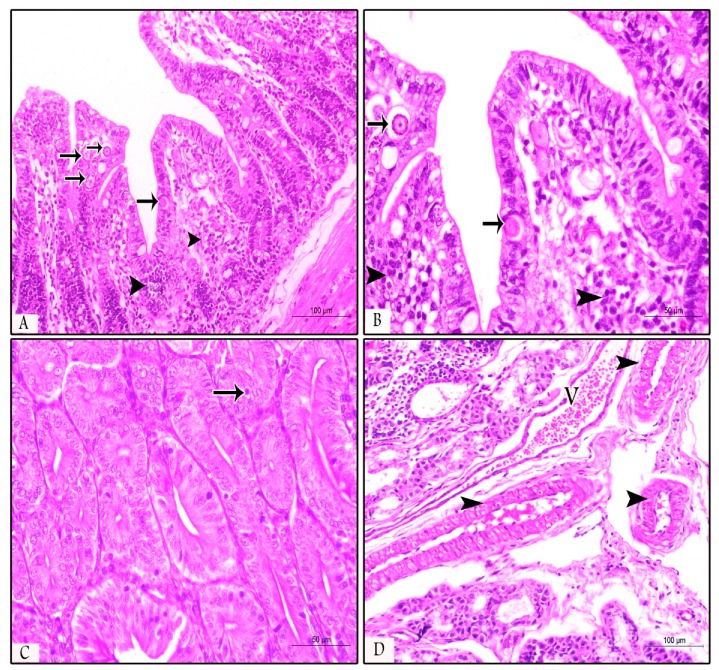
Jejunum of the PS-P group (prebiotic supplemented) showing: (**A**) Intact intestinal epithelium with few parasitic stages (arrows) and mononuclear cell infiltration in the lamina propria (arrow heads, H&E, ×200); (**B**) higher magnification of the intestinal mucosa showing parasitic stages, including gametocytes and oocysts (arrows) and leucocytic infiltration (arrow heads, H&E, ×400); (**C**) intestinal glands contain one coccidian stage (arrow, H&E, ×400); and (**D**) moderate congestion of blood vessel (V) and mild congestion of the other vessels (arrow heads, H&E, ×200).

**Figure 5:**
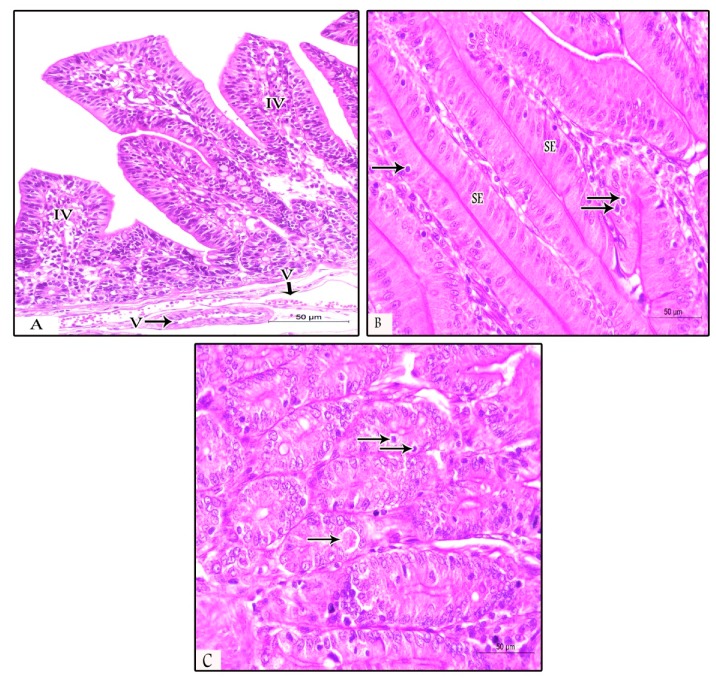
Different parts of the intestine in the PS-T (prebiotic treated) group: (**A**) Jejunum with an intact absorptive surface epithelial lining, intestinal villi (IV), mononuclear cell infiltration, intestinal glands (G), and mild congestion of blood vessels (V) (H&E, ×400); (**B**) duodenal villi lined with columnar epithelium (SE) with few parasitic stages (arrows, H&E, ×400); and (**C**) intestinal glands of the ileum showing degenerated parasitic stages (arrows, H&E, ×400).

The examined samples (ileum, duodenum, and jujenum) from prophylactic and therapeutic experiments showed differences in the number of parasitic stages. The count of developing stages was significantly higher in the NI-P group than in the other groups, with nearly similar counts in the PS-P and PS-T groups. The histopathological examination of the examined tissues in the NI -P group revealed severe inflammatory changes, massive infiltration of mononuclear cells, and sloughing of the absorptive epithelium (Figure 2A). Additionally, the sites of the intestinal absorptive epithelium were occupied by a huge number of different developmental stages of *Eimeria* spp. (Figure 2B,C). Focal areas of discrete hemorrhages were also detected (Figure 2B). The number of absorptive epithelia and goblet cells were markedly diminished at the sites of the coccidial stages, and disappeared in areas of massive infiltration of developing stages of *Eimeria* spp. Some developmental stages of *Eimeria* spp. were observed in the lamina propria (Figure 2D) and the glandular epithelium (Figure 2E) of different parts of the intestine. The submucosal blood vessels showed severe congestion (Figure 2F). In the NN-P group, the intestine showed normal architecture, intestinal villi, lamina propria, submucosa, and tunica muscularis (Figure 3A). The intestinal villi appeared normal, and their lining absorptive epithelia were devoid of any developing stages of *Eimeria* spp. (Figure 3B). The intestinal glands (crypts of leiberkhun) displayed normal architecture (Figure 3C). The intestine of rabbits in the PS-P group revealed mild histopathological changes, with an intact absorptive epithelial lining (Figure 4A). Few numbers of developmental stages of *Eimeria* spp. were reported within the epithelium (Figure 4B) and intestinal glands (Figure 4C). Areas of hemorrhagic foci were not detected except mild congestion of some submucosal blood vessels (Figure 4D). In the PS-T group, the intestine showed an intact surface epithelium lining, and the intestinal villi, with mild congestion of submucosal blood vessels (Figure 5A). Few numbers of developmental stages of *Eimeria* spp. were observed in the simple columnar absorptive epithelial lining of the intestinal villi (Figure 5B). The intestinal glands showed degenerated parasitic stages (Figure 5C). 

### 3.4. Biochemical Analysis

Both ALT and AST showed significantly higher values in the PS-P and NN-P groups than in the NI-P group (*p* ≤ 0.05). No differences in ALP, creatinine, total protein, and cholesterol were recorded among the different groups (Table 5 and Table 6). Additionally, these parameters remained within normal levels. 

## 4. Discussion

Rabbits are highly susceptible to the enteric pathogens, mainly in the early weaning period, which may be attributed to the unestablished intestinal microbiota, a less developed digestive system, and change in gut PH [10]. Coccidiosis is the most serious problem in rabbit farms causing high morbidity and mortality rates among all ages, especially in the young rabbits [3,29]. The European Union has banned the use of antibiotics as feed additives for growth promotion in animals since 2005 [30]. Currently, great efforts are directed toward replacing the antibiotics with alternative anticoccidials, including prebiotics and probiotics, which have beneficial effects on the host by stimulating the immune system, improving the productivity and performance in addition to their bactericidal and/or bacteriostatic activities. According to Ashayerizadeh et al. [31] prebiotics are growth promoters, which can be used as safe, alternative feed additives because they are able to improve growth of broiler chickens [32]. Nowadays, researchers have paid great attention to replace the commercial anticoccidial drugs with natural products due to the development of drug resistance [32]. Falcao-e- Cunha et al. [33] reported that prebiotics could prevent the adhesion of pathogens to the intestinal mucosa and stimulate the immune responses in rabbits. In the current study, dietary supplementation of prebiotic (Bio-Mos^®^) was applied for the prophylactic and therapeutic use of prebiotics against rabbit coccidiosis caused by the *Eimeria* parasite. In the prophylactic experiments, data showed a significant (*p* ≤ 0.05) reduction in fecal oocyst counts in the PS-P group, with body weight and weight gain remained relatively unchanged. However, the results of the therapeutic experiment showed a significant (*p* ≤ 0.05) decrease in fecal oocyst counts in the PS-T group when compared with the UI-T group. The body weight loss continued in the UI-T group and became negligible in the PS-T group at day seven PPS. These results are similar to those reported by Faber et al. [34] who found that *E. acervulina* affect the body weight in the broilers supplemented with a prebiotic product [34]. As natural biological response modifiers and promoters, prebiotics have the ability to increase host defense mechanism against infections [12]. Similarly, it was recently demonstrated that prebiotics could enhance the intestinal health, inhibit the epithelial invasion by pathogens and mucosal adherence of pathogens, and help to produce antimicrobial substances and/or stimulate mucosal immunity [35]. Furthermore, Roberfroid et al. [36] mentioned that prebiotics exhibit health promoting properties to host through the selective growth due to improving the nutrient digestibility [37,38]. Previously, prebiotics could prevent enteric diseases of rabbits by boosting gut colonization, modulate microbial community, and regulate production of cytokines and antibodies, and improve gut development and the overall broiler health [39,40]. Moreover, prebiotics inhibit the development of schizonts by stimulating the local immune mechanisms. This potential anticoccidial activity of prebiotic (MOS) appeared in the reduction of oocyst counts in the prebiotic-treated chickens [41,42]. However, the prebiotics are still unsuccessful in controlling *E. maxima* and *E. tenella* infections in broilers [43]. Generally, the prebiotic supplementation preserved the body weight in the prebiotic-treated rabbits. This finding may be attributed to the fact that the prebiotic constituents (mainly yeast derivatives) improve nutrient digestibility and intestinal villus length that lead to the increase of the absorptive surface in the intestine [44,45]. Histopathological findings in the duodenum, jujenum, and ileum in all groups revealed that different parasitic stages of *Eimeria* spp. were significantly higher in the NI-P group than in the UI-T group. However, the count of the developing stages of *Eimeria* spp. was nearly similar in both prophylactic (PS-P) and therapeutic (PS-T) experiments.

Our results were in agreement with those obtained by Yakhkeshi et al. [46] and Oso et al. [47] who described improved morphological parameters in the rabbit ileum (increased villus length) after prebiotic supplementation [46,47]. Additionally, the intestinal mucosa in the PS-P group was not as severely affected as in the NI-P group. This may be due to the positive effect of a prebiotic supplement on the intestinal villi [48,49]. 

Biochemical parameters, including ALT, ALP, AST, creatinine, total protein, and cholesterol were within the normal ranges [50]. Interestingly, prebiotic supplementation did not have any adverse effects on liver and kidney functions, total protein, and cholesterol. These findings are in agreement with previous reports [19,28].

## 5. Conclusions

Prophylactic supplementation of rabbits after weaning with a prebiotic preparation (Bio-Mos^®^) can minimize the adverse effect of intestinal coccidiosis in rabbits. 

## Figures and Tables

**Table 1:** Effect of prebiotic supplementation on oocyst count per gram (OPG) in experimentally infected rabbits.

Group	5 DPI	7 DPI	9 DPI	10 DPI
NN-P *	0.00 ± 0.00 ^c^	0.00 ± 0.00 ^c^	0.00 ± 0.00 ^c^	0.00 ± 0.00 ^c^
NI-P **	269 × 10^3^ ± 50.78 ^a^	175.83 × 10^3^ ± 38.68 ^a^	157.33 × 10^3^ ± 43.91 ^a^	130.83 × 10^3^ ± 43.38 ^a^
PS-P ***	97.33 × 10^3^ ± 19.63 ^b^	83.66 × 10^3^ ± 15.47 ^b^	77.83 × 10^3^ ± 15.39 ^b^	57.33 × 10^3^ ± 2.84 ^b^

Data presented as means and standard error of the mean (Mean ± SE). ^a–c^ means within the same column with different superscripts are significantly different at (*p* ≤ 0.05). DPI stands for days post-infection. * NN-P = non-supplemented non-infected control. ** NI-P = non-supplemented infected control. *** PS-P = prebiotic supplemented.

**Table 2:** Body weight (g) in the prophylactic experiment during infection in different groups of rabbits.

Group	Day 0 (Prebiotic Supplementation)	10 DPPS	10 DPI	WEIGHT Gain at End of Experiment
NN-P *	820.00 ± 64.29 ^a^	1000.33 ± 3.33 ^a^	1080.33 ± 3.33 ^a^	80.00 ± 0.00 ^a^
NI-P **	810.00 ± 37.85 ^a^	900.66 ± 4.66 ^b^	870.66 ± 6.66 ^c^	−30.00 ± 8.81 ^b^
PS-P ***	846.66 ± 63.85 ^a^	980.33 ± 2.88 ^a^	970.33 ± 31.79 ^b^	−10.00 ± 3.33 ^b^

Data are presented as means and standard error of the mean (Mean ± SE). ^a,b,c^ means within the same column with different superscripts are significantly different at (*p* ≤ 0.05). DPPS stands for days post-prebiotic supplementation. * NN-P = non-supplemented non-infected control. ** NI-P = non-supplemented infected control. *** PS-P = prebiotic supplemented.

**Table 3:** Oocysts count per gram of feces (OPG) during prebiotic treatment in naturally *Eimeria*-infected rabbits.

Group	0 DPPT	2 DPPT *	3 DPPT	4 DPPT	5 DPPT	7 DPPT
PS-T **	146.33 × 10^3^ ± 29.42	97 × 10^3^ ± 23.43	67 × 10^3^ ± 16.37	30.66 × 10^3^ ± 12.44	31.66 × 10^3^ ± 21.85	4 × 10^3^ ± 0.00 ^b^
UI-T ***	146 × 10^3^ ± 30.51	90.66 × 10^3^ ± 12.57	70 × 10^3^ ± 18.33	47.33 × 10^3^ ± 14.11	39.33 × 10^3^ ± 12.34	32 × 10^3^ ± 7.54 ^a^

Data are presented as means and standard error of the mean (Mean ± SE). ^a,b^ means within the same column with different superscripts are significantly different at (*p* ≤ 0.05). * DPPT stands for days post- prebiotic treatment. ** PS-T = prebiotic treated. *** UI-T = untreated infected.

**Table 4:** Therapeutic efficacy of prebiotic supplementation in body weight (g) of naturally *Eimeria*-infected rabbits.

Group	0 DPPT	7 DPPT *	Weight Gain at End of Experiment
PS-T **	751.66 ± 28.91	756.66 ± 30.32	6.00 ± 2.88
UI-T ***	733.33 ± 8.81	706.66 ± 6.66	−26.66 ± 8.33

Data are presented as means and standard error of the mean (Mean ± SE). * DPPT stands for days post-prebiotic treatment. ** PS-T = prebiotic treated. *** UI-T = untreated infected.

**Table 5:** Parasitic stage counts in the ileum, jejunum, and duodenum in all groups in both experiments (Prophylactic and therapeutic).

Group	Ileum	Duodenum	Jejunum
NN-P	0.00 ± 0.00 ^c^	0.33 ± 0.33 ^c^	0.66 ± 0.33 ^d^
NI-P *	18.33 ± 2.02 ^a^	23 ± 1.15 ^a^	35 ± 1.15 ^a^
PS-P **	7.00 ± 1.20 ^b^	5.33 ± 0.88 ^b^	7.66 ± 0.88 ^c^
PS-T ***	7.33 ± 1.20 ^b^	6.33 ± 0.88 ^b^	8.66 ± 1.45 ^c^
UI-T ****	16.00 ± 2.08 ^a^	20.33 ± 1.45 ^a^	30.66 ± 1.20 ^b^

Data are expressed as Mean ± SE. ^a–d^ mean within the same column with different superscripts are significantly different (*p* ≤ 0.05). * NI-P = non-supplemented infected control. ** PS-P = prebiotic supplemented. *** PS-T = prebiotic treated. **** UI-T = untreated infected.

**Table 6:** Biochemical parameters in the prophylactic experiment at day 10 post-infection.

Group	Total Protein	ALT	AST	ALP	Total Cholesterol	Createnine
NN-P *	6.42 ± 0.57	50.33 ± 3.17 ^b^	41.00 ± 4.04 ^b^	69.33 ± 28.58 ^a^	68.66 ± 14.43 ^a^	0.92 ± 0.18 ^a^
NI-P **	6.39 ± 0.24	67.66 ± 8.56 ^b^	103.00 ± 6.42 ^a^	73.00 ± 7.00 ^a^	91.00 ± 11.54 ^a^	0.95 ± 0.25 ^a^
PS-P ***	5.78 ± 0.29	105.66 ± 7.53 ^a^	37.66 ± 7.88 ^b^	63.33 ± 14.52 ^a^	66.33 ± 12.71 ^a^	0.72 ± 0.14 ^a^

Data presented as means and standard error of the mean (Mean ± SE). ^a,b^ mean within the same column with different superscripts significantly different at (*p* ≤ 0.05). *NN-P = non-supplemented non-infected control. **NI-P = non-supplemented infected control. *** PS-P = prebiotic supplemented.
